# Partially Defatted *Tenebrio molitor* Larva Meal in Diets for Grow-Out Rainbow Trout, *Oncorhynchus mykiss* (Walbaum): Effects on Growth Performance, Diet Digestibility and Metabolic Responses

**DOI:** 10.3390/ani10020229

**Published:** 2020-01-31

**Authors:** Giulia Chemello, Manuela Renna, Christian Caimi, Inês Guerreiro, Aires Oliva-Teles, Paula Enes, Ilaria Biasato, Achille Schiavone, Francesco Gai, Laura Gasco

**Affiliations:** 1 Department of Agricultural, Forest and Food Sciences, University of Turin, Largo P. Braccini 2, 10095 Grugliasco (TO), Italy; giulia.chemello@unito.it (G.C.); christian.caimi@unito.it (C.C.); ilaria.biasato@unito.it (I.B.); laura.gasco@unito.it (L.G.); 2 Department of Veterinary Sciences, University of Turin, Largo P. Braccini 2, 10095 Grugliasco (TO), Italy; achille.schiavone@unito.it; 3 CIMAR/CIIMAR-Centro Interdisciplinar de Investigação Marinha e Ambiental, Universidade do Porto, Terminal de Cruzeiros do Porto de Leixões, Av. General Norton de Matos s/n, 4450-208 Matosinhos, Portugal; imsguerreiro@gmail.com (I.G.); aoteles@fc.up.pt (A.O.-T.); enes.ciimar@gmail.com (P.E.); 4 Departamento de Biologia, Faculdade de Ciências, Universidade do Porto, Rua do Campo Alegre s/n, Ed. FC4, 4169-007 Porto, Portugal; 5 Institute of Science of Food Production, National Research Council, Largo P. Braccini 2, 10095 Grugliasco (TO), Italy; francesco.gai@ispa.cnr.it

**Keywords:** defatted insect meal, yellow mealworm, carnivorous fish, productive traits, apparent digestibility coefficient, hepatic enzyme

## Abstract

**Simple Summary:**

The current developments in the aquaculture sector have highlighted the need to find sustainable ingredients to replace fishmeal as a protein source in fish feeds. The use of insect meals may be a valid option, due to their good nutritional values and low ecological footprint. In this study, we evaluated the effects of a progressive fishmeal substitution with increasing concentrations of a partially defatted yellow mealworm meal in rainbow trout diets. We observed that the total substitution of fishmeal with insect meal is feasible and that there are no negative effects on fish growth or on the digestibility of most nutrients. The activities of hepatic enzymes involved in the amino acid metabolism and lipid synthesis were also evaluated. The enzymatic activities were not negatively influenced by insect meal inclusion in the diets. These results are of practical application for feed manufacturers and farmers, as they support the inclusion of insect meals in fish diets to obtain sustainable feeds that able to support an increase in aquaculture production.

**Abstract:**

Insect meals are good candidates to replace fishmeal as new protein sources in aquafeeds. This study evaluated the effects of fishmeal replacement with different dietary inclusion levels of a partially defatted *Tenebrio molitor* (L.) larva meal (TM) on rainbow trout (*Oncorhynchus mykiss* Walbaum) growth, diet digestibility, and hepatic intermediary metabolism. A 154-day growth trial was performed with 252 rainbow trout (78.3 ± 6.24 g) randomly divided into twelve tanks and fed four experimental diets containing increasing levels of TM: 0% (TM0), 25% (TM25), 50% (TM50), and 100% (TM100) of fishmeal substitution, corresponding to TM dietary inclusion levels of 0%, 5%, 10%, and 20%, respectively. A digestibility trial was performed feeding 180 rainbow trout (94.6 ± 7.31 g) with the experimental diets used in the growth trial. The growth parameters were not affected by TM dietary inclusion. Regarding the evaluated apparent digestibility coefficients (ADC), only the ADC of crude protein was affected, showing the following trend: TM0 = TM25 > TM50 > TM100. The activities of key hepatic amino acid catabolic and lipogenic enzymes were not affected by the dietary composition. The results suggest that a partially defatted TM could totally replace fishmeal in commercial rainbow trout diets without negative effects on fish performance.

## 1. Introduction

Aquaculture production is growing faster than any other major food sector. Indeed, with 110.2 million tons harvested in 2016, it will provide the most reliable supply of seafood in the upcoming years [1]. Fish diets, and those for carnivorous species in particular, have traditionally incorporated a large amount of fishmeal (FM), which represents a high-quality source of protein with a well-balanced essential amino acids (EAA) and fatty acid (FA) profile, high digestibility, and good palatability [2].

FM production depends on the catches of small pelagic wild stocks such as menhaden, herring, anchovies and sardines, which are processed to obtain different products [3]. Unfortunately, the unrestrained use of FM over the last few decades has put wild stocks under critical pressure with no prospect of rapid recovery [4]. As a result, the aquaculture industry has to face the problem of a limited FM supply and a consequent increase in its cost. Over the last few years, researchers and feed manufacturers have focused their efforts on reducing FM inclusion levels in commercial fish diets while, at the same time, maintaining fish health and the nutritional quality of the final products.

Many advances have been made in the partial replacement of FM with alternative protein sources in aquafeeds [5]. The amount of FM used in diets for carnivorous species has shown a clear decreasing trend toward a more selective use of FM as a strategic ingredient at lower levels, depending on the fish life-cycle stage and species of the fish [6]. Likewise, the amount of FM in feeds for omnivorous fish has also been reduced, especially in grow-out feeds [5]. However, overall, the use of FM use in the aquafeed sector has continued to increase as a consequence of the growth in aquaculture production and the related consumption of aquafeeds [5]. A further reduction in FM inclusion in aquafeeds is thus mandatory.

Plant protein sources are the most common alternatives used to replace FM. Unfortunately, they have shown adverse effects, such as an extremely variable protein content, EAA imbalances, and anti-nutritional factors, which limit their use in diet formulations [6]. Insects have recently been considered promising alternative protein candidates to substitute FM in aquafeeds, thanks to their interesting nutritional values, in terms of their balanced amino acid (AA) profile, and their lipid, vitamin and mineral contents [7]. Interest in insects as an innovative aquafeed ingredients has grown rapidly within the scientific community and among stakeholders and their use in aquafeeds was approved by the European Commission (Annexe II of Regulation 2017/893 of 24th May 2017), which authorized the use of insect-derived processed animal proteins from seven insect species (two flies, two mealworms and three crickets). Compared to conventional livestock, the rearing of insects to produce animal feeds offers several ecological and economic advantages, because insects grow and reproduce easily, generate low greenhouse gas emissions and can be reared on discarded organic by-products [8,9]. Moreover, rearing insects on bio-waste and organic side streams meets the recycling principles of the circular economy, thus reflecting the efforts of the EU to develop a sustainable, resource-efficient, low carbon and competitive economy [10,11].

Yellow mealworm, *Tenebrio molitor* (L.) is one of the seven insect species authorized by the European Union. It is a worldwide distributed beetle; and its larvae can easily be reared on low-nutritive plants and can efficiently convert food waste and agricultural by-products into high-quality biomass [12]. They are rich in proteins (44.1%–60.3% on a dry matter (DM) basis) and lipids (16.6%–43.1% DM) and their AA and FA profiles make them suitable for their inclusion in animal feeds [13].

The use of *Tenebrio molitor* larvae meal (TM) as a partial substitute for conventional protein sources has been studied for different aquaculture species, and promising results have been observed for fish growth performance, diet digestibility, and immune system parameters [14,15,16,17,18,19,20,21]. However, in the majority of these studies, the experimental diets were characterized by just a few ingredients (fewer than those normally used in commercial fish diets formulations) and by high levels of FM inclusion (up to 75% as fed). Thus, these diets are not truly representative of the commercial diets currently used in aquaculture.

It should also be considered that, over the last few years, insect manufacturers have increased the production of defatted insect meals. The defatting process allows insect meals to be obtained with larger amounts of crude protein (CP) and better resistance to degradation than full-fat insect meals. Indeed, the latter contain a high lipid content, which in turn makes the extrusion process difficult. Therefore, a defatting process could provide a useful product to reach an adequate feed composition [22]. To date, only a few studies have been performed to evaluate the use of defatted TM in the diets of different fish species [23,24]. Further investigations should be performed to assess the effects of partially defatted TM dietary inclusion in commercial diets.

The present research was designed to assess the effects of a progressive FM substitution (0%, 25%, 50% and 100%) with a partially defatted TM (corresponding to dietary inclusion levels of 0%, 5%, 10% and 20%) in commercial diets on the growth performance, somatic indexes, nutrient digestibility and liver activity of key enzymes of lipogenic and amino acid catabolic pathways in grow-out rainbow trout, *Oncorhynchus mykiss* (Walbaum).

## 2. Materials and Methods

A growth trial and a digestibility trial were conducted at the Experimental Facility of the Department of Agricultural, Forest and Food Sciences (DISAFA) of the University of Turin (Italy). The experimental protocol was designed according to the guidelines of the current European and Italian laws on the care and use of experimental animals (European directive 86 609/EEC, put into law in Italy with D.L. 116/92). The experimental protocol was approved by the Ethical Committee of DISAFA (protocol n° 143811).

### 2.1. Experimental Diets

The used TM was supplied by Ÿnsect (Evry, France). The larvae had been raised on plant by-products and partially defatted using a mechanical process. No other information was given by the producer about either the rearing substrate or the processing methodologies, as this information is considered confidential.

Four experimental diets were formulated, in accordance with SPAROS LDA (Olhão, Portugal) and the TM producer, to be isonitrogenous (CP: about 42.5% as fed), isolipidic (ether extract (EE): about 24.2% as fed), and isoenergetic (gross energy (GE): about 23.8 MJ/kg). The diets were prepared including, as fed basis, increasing levels of a partially defatted TM in substitution of 0% (TM0), 25% (TM25), 50% (TM50) and 100% (TM100) of FM, corresponding to dietary inclusion levels of TM equal to 0%, 5%, 10% and 20%, respectively. In order to ensure that the experimental diets remained isonitrogenous, isolipidic and isoenergetic, and because of the different chemical compositions of TM and FM, the amounts of some other dietary ingredients (i.e., wheat gluten, wheat meal and sardine oil) were modified slightly with the dietary increase in TM inclusion. Moreover, AA supplementation was included in the diet formulations to meet the EAA requirements of the fish.

In order to prepare the experimental extruded diets (SPAROS LDA), all the powder ingredients were mixed according to the target formulation in a double-helix mixer (500L, TGC Extrusion, France) and ground (below 400 µm) in a micropulverizer hammer mill (SH1, Hosokawa-Alpine, Germany). The diets (pellet size: 4.0 mm) were manufactured using a twin-screw extruder (BC45, Clextral, France) with a screw diameter of 55.5 mm. The extruded pellets were dried in a vibrating fluid bed dryer (DR100, TGC Extrusion, France). After cooling, oils were added by means of vacuum coating (PG-10VCLAB, Dinnissen, The Netherlands). Immediately after coating, the diets were packed in sealed plastic buckets and shipped to the research site.

The composition of the ingredients of the experimental diets is shown in Table 1.

### 2.2. Chemical Analyses of Feed

Feed samples were ground using a cutting mill (MLI 204; Bühler AG, Uzwil, Switzerland) and analyzed for DM (AOAC #934.01), CP (AOAC #984.13) and ash (AOAC #942.05) contents according to AOAC International [25]; EE (AOAC #2003.05) was analyzed according to AOAC International [26]. The GE content was determined using an adiabatic calorimetric bomb (C7000; IKA, Staufen, Germany). The proximate composition of the experimental diets is shown in Table 2. Chitin was estimated according to Finke [27] by correction considering the AA content of the acid detergent fiber (ADF) fraction and assuming that the remainder of the ADF fraction was chitin. The FA composition analysis of the experimental diets was performed as reported in Renna et al. [28]. The fatty acid methyl esters (FAME) were separated, identified and quantified as reported by Ravetto Enri et al. [29]. The results reported in Table 3 are expressed as mg/100 g DM.

All the chemical analyses of the feeds were performed in duplicate.

### 2.3. Growth Trial

#### 2.3.1. Fish and Rearing Conditions

Two hundred and fifty-two grow-out rainbow trout purchased from a private fish hatchery (“Troticoltura Bassignana”; Cuneo, Italy) were used to carry out a 154-day trial after a two-week period of acclimation during which the fish were fed a commercial diet (42% CP and 22% EE, Skretting Italia Spa, Mozzecane (Vr), Italy).

At the beginning of the trial, the fish were anesthetized slightly (MS-222, PHARMAQ Ltd., UK; 60 mg/L), individually weighed (78.3 ± 6.24 g) and randomly distributed into twelve 400-L tanks (three replicate tanks per diet, twenty-one fish per tank). Artesian well water (constant temperature of 13 ± 1 °C) was supplied in a flow-through open system (tank water inflow: 8 L/min). The dissolved oxygen levels were measured every two weeks and they ranged between 7.6 and 8.7 mg/L. The fish were fed 1.6% of the tank biomass for the first 8 weeks and then, according to the fish growth and water temperature, the daily quantity of distributed feed was decreased to 1.4%. The fish were fed twice a day (08:00 and 15:00) six days per week and the feed intake was monitored at each administration. In order to update the daily feeding rate, the biomass tanks were weighed in bulk every 14 days. Mortality was checked every day.

#### 2.3.2. Growth Performance

At the end of the trial, the fish were left unfed for one day, anesthetized slightly (MS-222, PHARMAQ Ltd., Sandleheath, UK; 60 mg/L) and weighed individually (KERN PLE-N v.2.2; KERN and Sohn GmbH, Balingen-Frommern, Germany; d: 0.001). The following performance indexes were calculated:

Mortality (%) = 100 − [(number of dead fish/number of fish at start) × 100](1)

Individual weight gain (iWG, g) = iFBW (individual final body weight, g) − iIBW (individual initial body weight, g)(2)

Specific growth rate (SGR, % day^−1^) = [(lnFBW − lnIBW)/number of days] × 100 (3)

Feed conversion ratio (FCR) = total feed supplied (g, DM)/WG (g)(4)

Protein efficiency ratio (PER) = WG (g)/total protein fed (g, DM)(5)

Feed intake (FI) = total amount of feed consumed (g, DM)/((final number of fish + initial number of fish)/2)/days(6)

SGR, FCR, PER and FI were calculated per tank.

#### 2.3.3. Condition Factor and Somatic Indexes

Fifteen fish per treatment (five fish per tank) were killed by overanaesthesia (MS-222; PHARMAQ Ltd., UK; 500 mg/L). The fish were weighed individually (KERN PLE-N v.2.2; KERN and Sohn GmbH, Balingen-Frommern, Germany; d: 0.001) and the total length of the fish was measured to determine the Fulton’s condition factor (K). The fish were slaughtered to calculate the hepatosomatic index (HSI), the viscerosomatic index (VSI), and the coefficient of fatness (CF). The condition factor and the somatic indexes were calculated as follows:

K = [fish weight (g)/(body length)^3^ (cm)] × 100 (7)

HSI (%) = [liver weight (g)/fish weight (g)] × 100(8)

VSI (%) = [gut weight (g)/fish weight (g)] × 100(9)

CF (%) = [perivisceral fat weight (g)/fish weight (g)] × 100(10)

### 2.4. Digestibility Trial

An in vivo digestibility trial was performed to assess the apparent digestibility coefficients (ADCs) of the diets. One hundred and eighty rainbow trout (94.6 ± 7.31 g) were divided into twelve 250-L cylindroconical tanks connected to the same open water system as that of the growth trial (three replicate tanks per diet, fifteen fish per tank). After fourteen days of acclimation to the experimental diets, the fish were fed by hand to apparent visual satiety twice a day (08:00 and 15:00), six days per week. The ADCs were measured using the indirect acid-insoluble ash method. To this aim, 1% celite^®^ (Fluka, St. Gallen, Switzerland) was added to the diets as an inert marker in the substitution of 1% of wheat meal. The faeces were collected daily from each tank for four consecutive weeks, using a continuous automatic device (Choubert’s system), as described by Palmegiano et al. [30]. The feces were freeze-dried and frozen (−20 °C) until analyzed. The ADCs of the DM (ADC_DM_), CP (ADC_CP_), EE (ADC_EE_) and GE (ADC_GE_) were calculated as reported by Caimi et al. [31] and expressed as a percentage.

### 2.5. Hepatic Enzyme Activities

Liver samples were collected (nine replicates per treatment) and stored at −80 °C to measure the alanine aminotransferase (ALAT; EC 2.6.1.2), aspartate aminotransferase (ASAT; EC 2.6.1.1) and glutamate dehydrogenase (GDH; EC1.4.1.2) activities. The liver samples were homogenized (dilution 1:10) in an ice-cold buffer (30 mM 4-(2hydroxyethyl)-1-piperazineethanesulfonic acid (HEPES), 0.25 mM saccharose, 0.5 mM ethylenediaminetetraacetic acid (EDTA), 5 mM K_2_HPO_4_, 1 mM dithiothreitol (DTT); pH 7.4). After being centrifuged at 1000× *g* for 10 min at 4 °C, the supernatants were sonicated for 1 min (pulse 1 s, amplitude 50) and centrifuged again at 15,000× *g* for 20 min at 4 °C. The resultant supernatant was collected for enzyme activity measurements. GDH activity was measured using 10 mM of L-glutamic acid, as described by Bernt and Bergmeyer [32]. ALAT and ASAT were assayed using Spinreact kits (ALAT/GPT, ref. 41282; ASAT/GOT, ref. 41272) according to the manufacturer’s instructions.

For glucose-6-phosphate dehydrogenase (G6PD; EC 1.1.1.49), malic enzyme (ME; EC 1.1.1.40), and fatty acid synthetase (FAS; EC 2.3.1.38) activities, the liver samples were homogenized (dilution 1:5) in ice-cold buffer (0.02 M Tris-HCl; 0.25 M sucrose; 2 mM EDTA; 0.1 M sodium fluoride; 0.5 mM phenyl methyl sulphonyl fluoride (PMSF); 0.01 M β-mercapto ethanol; pH 7.4) and the homogenate was centrifuged at 30,000× *g* for 20 min at 4 °C. G6PD activity was measured according to Bautista et al. [33], ME activity was measured according to Ochoa [34] and FAS activity according to Chang et al. [35], as modified by Chakrabarty and Leveille [36].

All the enzyme activities were expressed per mg of hepatic soluble protein (specific activity). The protein concentration was determined according to Bradford [37] using the Sigma-Aldrich protein assay kit (ref. B6916) with bovine serum albumin as standard. One unit of enzyme activity was defined as the amount of enzyme that catalyzed the hydrolysis of 1 µmol of substrate per min at the assay temperature (37 °C). All the enzyme assays were carried out using a Multiskan GO microplate reader (Model 5111 9200; Thermo Scientific, Nanjing, China). All the reagents used for the enzymatic analysis were purchased from Sigma-Aldrich (Química, S.L., Sintra, Portugal).

### 2.6. Statistical Analyses

The obtained data were analyzed by means of one-way ANOVA, using IBM SPSS Statistics v. 25.0 for Windows. The following model was used:

Y_ij_ = μ + D_i_ + ε_ij_, (11)

where Y_ij_ = observation; μ = overall mean; D_i_ = effect of diet (TM0, TM25, TM50, TM100); ε_ij_ = residual error.

The assumption of normality was checked using the Kolmogorov–Smirnov test. Levene’s homogeneity of variance test was used to assess homoscedasticity. If such an assumption did not hold, the Brown–Forsythe statistic was applied to test the equality of group means instead of the F one. Pairwise multiple comparisons were performed to test the difference between each pair of means (Tukey’s test and Tamhane’s T2 in the cases of assumed or not assumed equal variances, respectively). The results were expressed as the mean and pooled standard error of the mean (SEM). Significance was set at *p* ≤ 0.05.

## 3. Results

### 3.1. Diets

The fish willingly accepted all the experimental diets and all the supplied feeds were consumed without rejection or loss. The proximate composition analyses conducted at the DISAFA laboratories revealed that all the analyzed parameters (DM, CP, EE and ash) were comparable among the experimental diets (Table 2), according to the formulation provided by the producer. The GE values were also verified to be similar for all the diets (about 22.56 MJ/kg as fed).

### 3.2. Growth Trial

#### 3.2.1. Growth Performance

Table 4 presents the mortality (%), growth performance and feed utilization of the fish fed the experimental diets. Mortality ranged from 7.94% (TM100) to 11.11% (TM0 and TM50) and was not significantly different among treatments (*p* > 0.05). At the end of the trial, the body weight of the fish from all the treatments had more than tripled. No significant differences among the treatments were observed for any of the considererd growth performance parameters (*p* > 0.05).

#### 3.2.2. Condition Factor and Somatic Indexes

No significant differences among treatments were observed for the condition factor or the somatic indexes. The only exception was HSI, which was higher (*p* < 0.05) in the fish fed diet TM100 than the fish fed diet TM0, while the fish fed diets TM25 and TM50 showed intermediate values (Table 5).

### 3.3. Digestibility Trial

ADC_CP_ showed the following decreasing trend with the increase in TM in the diets: TM0 = TM25 > TM50 > TM100. Moreover, ADC_DM_, ADC_EE_ and ADC_GE_ did not show any significant differences among treatments (Table 6).

### 3.4. Hepatic Enzyme Activities

The activity of both hepatic amino acid catabolic (ALAT, ASAT, and GDH) and lipogenic enzyme (G6PD, ME, and FAS) was not significantly affected by the dietary treatment (*p* > 0.05) (Table 7).

## 4. Discussion

### 4.1. Growth Performance

TM is considered a good alternative ingredient for the partial replacement of FM in the diets of several fish species [14,15,18,20]. However, high dietary inclusion levels (usually higher than 25%–30%) have been reported to negatively affect fish performance, usually due to a deficiency of some nutrients, such as some EAAs [7,13]. Unlike the results found in the literature, the results of the present study reveal that the inclusion of TM in rainbow trout diets did not affect either the fish growth performance or feed utilization for any of the considered inclusion levels. These results can be partly justified by considering the composition of the experimental diets. The control diet in the present study in fact had a low FM inclusion level (20%), in order to reflect the current levels used in commercial diets. Therefore, the full dietary replacement of FM (TM100 diet) corresponded to an inclusion level of TM equal to 20%. Such a TM inclusion level is much lower than the inclusion levels (25% and 50%) used in previous studies [16,20]. It could be argued that such a low dietary TM content was unable to affect rainbow trout growth negatively. Another concurrent reason for the lack of diet effect on trout growth could be the EAA supplementation of the experimental diets. Similarly, the growth performance of common yellow catfish (*Pelteobagrus fulvidraco* Richardson) juveniles was not affected by diets containing amounts of TM of up to 75% in replacement of FM, as demonstrated by Su et al. [18]. In addition, the control diet in the above-mentioned study was formulated with a low FM concentration (24% of inclusion) to reflect the levels in commercial diets for that species.

The present study is one the first to evaluate the effects of total FM replacement with partially defatted TM in rainbow trout diets. Rema et al. [23] have recently described the effects of total FM replacement with defatted TM larva meal in rainbow trout diets. The TM substitution levels were 20%, 30%, 60% and 100%—that is, corresponding to 5%, 7.5%, 15% and 25% of TM inclusion, respectively—and thus similar to those used in our study. Rema et al. [23] observed that the gradual dietary increase in the TM level led to a significant and progressive increase in FBW, SGR and PER, and to a significant reduction in FCR, when compared to the control diet. Conversely, no improvement in fish growth performance was observed in our study. However, it should be highlighted that Rema et al. [23] performed a trial with rainbow trout fingerlings (IBW: 5.01 g), while grow-out rainbow trout (IBW: 78.3 g) were used in our trial. Considering that the fish-specific growth rate decreases as body size increases and that the dietary fat content in the two studies was comparable, the substantial disparity in the initial weight of the fish could reliably explain the differences observed in the fish growth performance.

In agreement with the findings of Rema et al. [23], Ido et al. [24] observed that both the partial and total replacement of FM in diets with a defatted TM positively promoted the growth of red seabream (*Pagrus major* Temminck and Schlegel). WG increased in the fish fed a diet characterized by a 100% replacement of FM with TM, while FCR was not affected by the presence of insect meal in the diet. Therefore, these authors assumed that the TM diets were remarkably preferred by the fish [24].

Among the evaluated somatic indexes, the HSI was significantly higher in the fish fed the TM100 diet than the fish fed the TM0 diet. In contrast, Belforti et al. [14] observed a decrease in the HSI level in rainbow trout fed diets with increasing levels of full-fat TM. Considering that the experimental diets did not affect the fish WG, these authors related this outcome to a voluntary reduction in fish intake due to the high quantity of fat present in the full-fat TM that was used. It is well known that the liver is a key organ of the fish metabolism and that HSI is an index that is usually utilized to investigate the effect of diets on liver functionality [38]. HSI values that exceede a standard range (between 1% and 2%) could reflect disorders in the glucose and lipid metabolism, the existence of an oxidized feed, or even a vitamin deficiency [39]. In our study, the HSI values for all treatments fell within the normal physiological range. Therefore, the differences observed between fish fed the control diet and the TM100 diet were not supposed to have caused any negative effects on fish health.

Almost all insect meals are considered low in lysine and tryptophan, with TM also being limited in sulfur amino acids [13]. The absence of any adverse effects on fish growth observed in our study could also be related to the fact that all the experimental diets were supplemented with some EAA (L-Arginine, L-Lysine, L-Tryptophan and DL-methionine) in order to allow the EAA requirements of rainbow trout to be fully met.

### 4.2. Digestibility Trial

All the experimental diets presented high values of ADC, thereby supporting the positive growth performance herein observed.

The most relevant outcome was observed for ADC_CP_, which significantly decreased when the fish were fed diets supplemented with the two highest inclusion levels of TM. Indeed, the ADC_CP_ of the TM100 diet was significantly lower than that of the TM50 diets. The decrease in ADC_CP_ could be related to the increasing amount of chitin in the TM-based diets (Table 2). Belforti et al. [14] found comparable results for TM-fed rainbow trout while Renna et al. [40] found comparable results for black soldier fly (*Hermetia illucens*)-fed rainbow trout. The digestibility of insect protein has been reported to vary and to depend on the amount of protein linked to chitin that negatively influences protein digestion [27]. Nevertheless, it is known that some chitinase activity has been identified in the digestive tract of some fish species, thus suggesting that certain fish, such as marine carnivorous teleosts, may possess chitinase and degrade chitin [41]. On the contrary, chitinase activity is low or completely absent in rainbow trout [22], and this could explain the reduction in ADC_CP_ in the fish fed the highest inclusion levels of TM. However, it should be highlighted that the reduction in protein digestibility observed in our study, although statistically significant, was of low amplitude, and this may explain why it did not negatively affect the overall growth performance of the fish.

As previously shown by other authors [14,23,40], ADC_DM_, ADC_EE_, and ADC_GE_ were not impaired by the inclusion of insect meals in the diets of rainbow trout.

### 4.3. Hepatic Enzyme Activity

To the best of our knowledge, the effect of TM dietary inclusion on the hepatic intermediary metabolism of rainbow trout has not yet been reported in any published literature. TM dietary inclusion did not influence the activity of the AA catabolic enzymes, ALAT, ASAT, and GDH. The nutritional regulation of the AA metabolism has already been reviewed extensively [42,43,44]. It is well known that dietary protein levels exert little effect on the liver’s AA catabolism, whereas there is a relatively good response of these enzymes to AA intake [44]. As mentioned above, the experimental diets were supplemented with EAA to supply a balanced amino acid profile in the TM diets in order to be comparable to the FM-containing control diet. This could explain the absence of any significant differences in the ASAT, ALAT, and GDH activities among the diets. Similarly, a previous study performed on gilthead sea bream (*Sparus aurata* L.) demonstrated that partial or total substitution of FM with a mixture of plant protein sources balanced with EAA did not modify the hepatic activity of AA catabolic enzymes [45]. Furthermore, a recent study performed by Guerreiro et al. [46] observed that feeding meagre (*Argyrosomus regius* Asso) juveniles with increasing dietary levels of *Hermetia illucens* larva meal did not affect the activity of the hepatic amino acid catabolic enzymes.

Regarding lipogenic enzymes activities, several studies have demonstrated the primary role of fish liver in de novo FA synthesis [47,48,49,50]. Indeed, the activities of such hepatic lipogenic enzymes as FAS (a multi-enzyme complex which, together with acetyl-CoA carboxylase, catalyzes de novo FA synthesis), G6PD and ME (the suppliers of nicotinamide adenine dinucleotide phosphate (NADPH), which is essential for FAS activity) [51], have been evaluated extensively. It has already been demonstrated that the activity of these lipogenic enzymes is negatively affected when fish are fed high-fat diets [48]. Different FA compositions in fish diets could also affect the activity of lipogenic enzymes. In particular, dietary polyunsaturated omega-3 fatty acids (such as C20:5n3 or C22:6n3, together with high levels of C18:3n3) have been reported to inhibit FAS and G6PD activities in hepatocyte rainbow trout cultures and in gilthead sea bream, respectively [52,53]. In the present study, the activity of the hepatic lipogenic enzymes was not influenced by the dietary of TM inclusion, as the experimental diets were formulated to be isolipidic. Moreover, comparable levels of C18:3n3, C20:5n3 and C22:6n3 were found in the experimental diets.

As far as ME is concerned, previous studies have demonstrated that its activity is related to growth rate variations rather than to different dietary treatments [48,54]. In the current trial, SGR and the other growth indices did not vary among the experimental groups, thus explaining the similar ME activity among treatments.

## 5. Conclusions

This study has evaluated, for the first time, the effects of the dietary inclusion of a partially defatted *Tenebrio molitor* larva meal on the growth performance, diet digestibility, and hepatic intermediary metabolism of practical diets for on-growing rainbow trout. The obtained results show that, in the current typical commercial diets that contain about 20% of FM and a well-balanced EAA profile, FM could be substituted completely by TM, without any negative effects on fish growth, the condition factor or the activity of hepatic amino acid catabolizing and lipogenic enzymes. Among the digestibility coefficients, only ADC_CP_ was shown to be to be negatively affected by the inclusion of dietary insect meal, but it should also be highlighted that, in absolute values, the ADC remained high in all the treatments.

These results are of practical application for feed manufacturers and farmers. The inclusion of insect meals in fish diets could lead to sustainable feeds that would be able to support an increase in aquaculture production without the massive use of conventional protein sources, which are characterized by strong environmental impacts.

## Figures and Tables

**Table 1:** Ingredients (% as fed) of the experimental diets.

Ingredient	TM0	TM25	TM50	TM100
Fishmeal 65 (Peruvian)	20.00	15.00	10.00	0
*Tenebrio molitor* larva meal	-	5.00	10.00	20.00
Soy protein concentrate	18.00	18.00	18.00	18.00
Wheat gluten	7.75	7.40	7.40	7.06
Corn gluten	8.00	8.00	8.00	8.00
Soybean meal 48	7.00	7.00	7.00	7.00
Wheat meal	14.23	14.00	14.23	13.80
Sardine oil	4.30	4.26	4.20	4.10
Soybean oil	8.60	8.52	8.40	8.20
Rapeseed oil	8.60	8.52	8.40	8.20
Soy lecithin	0.50	0.50	0.50	0.50
Vit-Min Premix	1.00	1.00	1.00	1.00
Antioxidant	0.20	0.20	0.20	0.20
Sodium propionate	0.10	0.10	0.10	0.10
Monocalcium phosphate	0.52	0.92	0.92	1.72
L-Arginine	-	-	-	0.10
L-Lysine	-	0.30	0.30	0.60
L-Tryptophan	0.05	0.08	0.10	0.12
DL-methionine	0.15	0.20	0.25	0.30
Celite^®^	1.00	1.00	1.00	1.00

Abbreviations: TM, *Tenebrio molitor* larva meal.

**Table 2:** Proximate composition of the experimental diets (g/100 g as fed, unless otherwise stated).

Item	TM0	TM25	TM50	TM100
DM	93.77	93.83	94.13	94.41
CP	42.08	43.07	43.38	44.25
EE	22.63	22.95	22.44	22.36
Ash	7.57	7.09	6.49	5.60
Chitin	-	0.43	0.78	1.49
NFE ^1^	21.49	20.29	21.05	20.71
GE (MJ/kg as fed)	22.24	22.71	22.75	22.55

Abbreviations: DM, dry matter; CP, crude protein; EE, ether extract; NFE, nitrogen-free extract; GE, gross energy. ^1^ Calculated as 100 − [(100 − DM) + CP + EE + Ash + Chitin]. All values are reported as mean of duplicate analyses.

**Table 3:** Fatty acid composition (mg/100 g DM) of the experimental diets.

Item	TM0	TM25	TM50	TM100
C10:0	71.17	88.22	85.80	126.97
C14:0	303.11	296.23	299.63	309.09
C16:0	2418.11	2479.54	2517.68	2685.00
C16:1 *c*9	288.62	287.85	282.39	262.44
C18:0	651.44	674.13	674.11	734.35
C18:1 *c*9	6307.94	6654.64	6717.97	7095.02
C18:1 *c*11	448.42	465.82	457.72	434.23
C18:2 n6	5650.96	6007.19	6153.86	6517.83
C18:3 n3	1021.00	1058.46	1041.25	1049.31
C20:0	94.35	87.46	81.51	86.24
C20:1 *c*11	259.29	261.22	217.80	179.48
C20:2 n6	53.55	47.33	50.62	48.32
C20:3 n6	79.29	72.43	75.73	70.41
C20:5 n3	375.85	411.60	348.32	315.80
C22:0	48.13	51.95	52.19	54.66
C22:1 n9	95.98	84.80	56.26	12.00
C22:6 n3	102.36	139.92	159.95	101.11
Other FA ^1^	326.12	330.95	318.66	265.68
Σ SFA	3719.26	3812.03	3844.38	4112.18
Σ BCFA ^2^	62.23	63.04	62.74	63.55
Σ MUFA	7477.13	7831.43	7812.49	8052.37
Σ PUFA	7420.41	7877.44	7956.88	8204.94
Σ PUFA/Σ SFA	2.00	2.07	2.07	2.00
Σ n3 FA	1579.06	1688.61	1627.05	1525.86
Σ n6 FA	5841.34	6188.84	6329.83	6679.08
Σ n3/Σ n6 FA	0.27	0.27	0.26	0.23
TFA	18,616.80	19,520.90	19,613.74	20,369.49

Abbreviations: DM, dry matter; TM, *Tenebrio molitor* larva meal; *c*, *cis*; *t*, *trans*; FA, fatty acids; SFA, saturated fatty acids; BCFA, branchedchain fatty acids; MUFA, monounsaturated fatty acids; PUFA, polyunsaturated fatty acids; TFA, total fatty acids. ^1^ All less than 50 mg/100 g DM for each treatment: C12:0 + C15 *iso* + C15 *aiso* + C14:1 *c*9 + C15:0 + C16 *iso* + C17:0 + C17 *iso* + C17 *aiso* + C17:1 *c*9 + C18:1 *t* + C18:1 *c*12 + C18:1 *c*14 + C18:1 *t*16 + C18:3 n6 + C18:4 n3 + C20:1 *c*9 + C20:4 n6 + C22:5 n3. ^2^ C15 *iso* + C15 *aiso* + C16 *iso* + C17 *iso* + C17 *aiso*. All values are reported as mean of duplicate analyses.

**Table 4:** Mortality and growth performances of rainbow trout fed the control (TM0) and *Tenebrio molitor* larva meal (TM) experimental diets (n = 3).

Item	TM0	TM25	TM50	TM100	SEM	*p*-Value
Mortality (%)	11.11	9.52	11.11	7.94	2.332	0.965
iIBW (g)	78.24	78.19	78.34	78.25	0.259	0.224
iFBW (g)	390.48	421.90	408.81	431.69	11.822	0.699
IWG (g)	312.24	343.71	330.47	353.44	11.813	0.698
SGR (% day^−1^)	1.04	1.09	1.07	1.11	0.019	0.688
FCR	1.07	1.09	1.11	1.02	0.022	0.594
PER	2.09	2.00	1.96	2.09	0.040	0.652
FI (g DM fish^−1^ day^−1^)	2.69	2.44	2.84	2.97	0.096	0.242

Abbreviations: SEM, standard error of the mean; *p*, probability; iIBW, individual initial body weight; iFBW, individual final body weight; IWG, individual weight gain; SGR, specific growth rate; FCR, feed conversion ratio; PER, protein efficiency ratio; FI, feed intake.

**Table 5:** Condition factor (K) and somatic indexes of rainbow trout fed the control (TM0) and *Tenebrio molitor* larva meal (TM) experimental diets (*n* = 15).

Item	TM0	TM25	TM50	TM100	SEM	*p*-Value
K	1.17	1.21	1.17	1.24	0.013	0.153
HSI	0.90 b	1.08 ab	0.93 ab	1.11 a	0.027	0.008
VSI	12.66	12.60	12.96	12.60	0.190	0.895
CF	3.51	3.45	3.66	3.42	0.171	0.963

Abbreviations: SEM, standard error of the mean; *p*, probability; K, Fulton’s condition factor; HSI, hepatosomatic index; VSI, viscerosomatic index; CF, coefficient of fatness. Different letters within a row indicate significant differences (*p* ≤ 0.05).

**Table 6:** Apparent digestibility coefficients (ADC) of dry matter, crude protein, ether extract and gross energy of rainbow trout fed the control (TM0) and *Tenebrio molitor* larva meal (TM) experimental diets (*n* = 3).

Item	TM0	TM25	TM50	TM100	SEM	*p*-Value
ADC_DM_	94.69	94.43	94.32	94.16	0.197	0.108
ADC_CP_	98.48 a	98.50 a	97.98 b	97.25 c	0.145	0.000
ADC_EE_	98.84	98.45	98.36	98.33	0.182	0.212
ADC_GE_	96.71	97.34	96.59	96.15	0.163	0.179

Abbreviations: SEM, standard error of the mean; *p*, probability; ADC_DM_, apparent digestibility coefficient of dry matter; ADC_CP_, apparent digestibility coefficient of crude protein; ADC_EE_, apparent digestibility coefficient of ether extract; ADC_GE_, apparent digestibility coefficient of gross energy. Different letters within a row indicate significant differences (*p* ≤ 0.05).

**Table 7:** Hepatic amino acid catabolic and lipogenic enzyme activities (mU mg protein^−1^) in rainbow trout fed the control (TM0) and *Tenebrio molitor* larva meal (TM) experimental diets (*n* = 9).

Item	TM0	TM25	TM50	TM100	SEM	*p*-Value
Amino acid catabolizing enzymes						
ALAT	312.7	326.3	359.0	361.4	9.374	0.173
ASAT	635.0	487.0	522.4	634.3	22.655	0.069
GDH	38.0	33.4	36.7	37.2	1.166	0.531
Lipogenic enzymes						
G6PD	228.7	195.8	208.5	226.7	7.702	0.392
ME	78.0	63.4	65.5	76.8	2.518	0.071
FAS	4.5	5.3	3.8	4.5	0.231	0.169

Abbreviations: SEM, standard error of the mean; *p*, probability; ALAT, alanine aminotransferase; ASAT, aspartate aminotransferase; GDH, glutamate dehydrogenase; G6PD, glucose-6-phosphate dehydrogenase; ME, malic enzyme; FAS, fatty acid synthase.
